# Supplementary material for: Ritualized aggressive behavior reveals distinct social structures in native and introduced range tawny crazy ants
Source: PLoS One. 2019 Nov 22;14(11):e0225597. doi: 10.1371/journal.pone.0225597 (PMC6874334; doi:10.1371/journal.pone.0225597)
Supplement: S1 Table — (DOCX) [file pone.0225597.s002.docx]

Supporting Table 1: Locality information for all study sites. The number of nests sampled per site is also provided.

| **Country** | **Site ID^1^** | **Latitude** | **Longitude** | **Nests (#)** |
| --- | --- | --- | --- | --- |
| Argentina | Argentina Site 1 | -28.5382° | -57.1863° | 46 |
| Argentina | Argentina Site 2 | -28.6530° | -57.4294° | 19 |
| Argentina | Argentina Site 3 | -28.0474° | -58.1344° | 10 |
| Uruguay | Rivera D. 1 | -30.9833° | -55.5001° | 2 |
| Uruguay | Rivera D. 2 | -31.0013° | -55.5152° | 3 |
| Uruguay | Rivera D. 3 | -31.0040° | -55.5036° | 2 |
| Uruguay | Rivera D. 4 | -31.0365° | -55.4972° | 2 |
| Uruguay | Rivera D. 5 | -31.0425° | -55.4850° | 2 |
| Uruguay | Rivera D. 6 | -31.1104° | -55.4198° | 2 |
| Uruguay | Paysandu D. 1 | -31.4636° | -57.8978° | 2 |
| Uruguay | Rivera D. 7 | -31.5990° | -54.9880° | 3 |
| Uruguay | Tacuarembo D. 1 | -32.1079° | -54.6701° | 2 |
| Uruguay | Cerro Largo 1 | -32.2899° | -54.8008° | 2 |
| Uruguay | Rio Negro D. 1 | -32.9881° | -58.0514° | 5 |
| USA | TX - Brazoria C. 1 | 29.1335° | -95.6215° | 2 |
| USA | TX - Wharton C. 1 | 29.2257° | -96.3111° | 2 |
| USA | TX - Bexar C. 1 | 29.4382° | -98.6426° | 2 |
| USA | TX - Hays C. 1 | 30.0702° | -97.8497° | 2 |
| USA | TX - Travis C. 4 | 30.2040° | -97.6975° | 2 |
| USA | TX - Travis C. 3 | 30.2170° | -97.8470° | 2 |
| USA | TX - Travis C. 2 | 30.4255° | -98.0413° | 2 |
| USA | TX - Travis C. 1 | 30.4481° | -97.7284° | 2 |
| USA | FL - Alachua C. 1 | 29.6227° | -82.4736° | 2 |
| USA | FL - Alachua C. 2 | 29.6306° | -82.4717° | 2 |

^1^ All Argentina sites are in Corrientes Province. Argentina Site 1 was in the town of Carlos Pelligrini located within the Iberá Provincial Reserve. Argentina Site 2 was on the private reserve, Estancia Rincón del Socorro. Site 3 was in Mburucuyá National Park. Abbreviations: D. - Department, C. - County, TX - Texas, FL – Florida.
