# Supplementary material for: Ritualized aggressive behavior reveals distinct social structures in native and introduced range tawny crazy ants
Source: PLoS One. 2019 Nov 22;14(11):e0225597. doi: 10.1371/journal.pone.0225597 (PMC6874334; doi:10.1371/journal.pone.0225597)
Supplement: Attachment — Submitted filename: Response to Reviewers.docx [file pone.0225597.s004.docx]

Dear PLOS Editorial Staff,

Please find below my point-by-point response to comments of the academic editor and reviewer. Many thanks for your efforts on this manuscript.

“To enhance the reproducibility of your results, we recommend that if applicable you deposit your laboratory protocols in protocols.io, where a protocol can be assigned its own identifier (DOI) such that it can be cited independently in the future.”

I have uploaded a detailed protocol for how to conduct aggression assays. Protocol DOI: dx.doi.org/10.17504/protocols.io.8z9hx96.

“Journal Requirements:

1. When submitting your revision, we need you to address these additional requirements.

 Please ensure that your manuscript meets PLOS ONE's style requirements, including those for file naming.”

I have reviewed the style templates and made all necessary changes in the document to match.

2.  We note that Figure [1] in your submission contains a map image which may be copyrighted. All PLOS content is published under the Creative Commons Attribution License (CC BY 4.0), which means that the manuscript, images, and Supporting Information files will be freely available online, and any third party is permitted to access, download, copy, distribute, and use these materials in any way, even commercially, with proper attribution. For these reasons, we cannot publish previously copyrighted maps or satellite images created using proprietary data, such as Google software (Google Maps, Street View, and Earth). For more information, see our copyright guidelines: <http://journals.plos.org/plosone/s/licenses-and-copyright>.

This is not a copyrighted image. This map image was generated in Arc GIS using our data for the purpose of this publication. It is fine if this is published under the Creative Commons Attribution License. To clarify this, I have added a statement to the methods citing the software used to generate the map.

We require you to either (1) present written permission from the copyright holder to publish these figures specifically under the CC BY 4.0 license, or (2) remove the figures from your submission:

 1.    You may seek permission from the original copyright holder of Figure(s) [1] to publish the content specifically under the CC BY 4.0 license.

 We recommend that you contact the original copyright holder with the Content Permission Form (<http://journals.plos.org/plosone/s/file?id=7c09/content-permission-form.pdf>) and the following text:

“I request permission for the open-access journal PLOS ONE to publish XXX under the Creative Commons Attribution License (CCAL) CC BY 4.0 (<http://creativecommons.org/licenses/by/4.0/>). Please be aware that this license allows unrestricted use and distribution, even commercially, by third parties. Please reply and provide explicit written permission to publish XXX under a CC BY license and complete the attached form.”

 Please upload the completed Content Permission Form or other proof of granted permissions as an "Other" file with your submission.

In the figure caption of the copyrighted figure, please include the following text: “Reprinted from [ref] under a CC BY license, with permission from [name of publisher], original copyright [original copyright year].

 This is not a copyrighted image. See above.

2.    If you are unable to obtain permission from the original copyright holder to publish these figures under the CC BY 4.0 license or if the copyright holder’s requirements are incompatible with the CC BY 4.0 license, please either i) remove the figure or ii) supply a replacement figure that complies with the CC BY 4.0 license. Please check copyright information on all replacement figures and update the figure caption with source information. If applicable, please specify in the figure caption text when a figure is similar but not identical to the original image and is therefore for illustrative purposes only.

The following resources for replacing copyrighted map figures may be helpful:

 USGS National Map Viewer (public domain): <http://viewer.nationalmap.gov/viewer/>

The Gateway to Astronaut Photography of Earth (public domain): <http://eol.jsc.nasa.gov/sseop/clickmap/>

Maps at the CIA (public domain): <https://www.cia.gov/library/publications/the-world-factbook/index.html> and <https://www.cia.gov/library/publications/cia-maps-publications/index.html>

NASA Earth Observatory (public domain): <http://earthobservatory.nasa.gov/>

Landsat: <http://landsat.visibleearth.nasa.gov/>

USGS EROS (Earth Resources Observatory and Science (EROS) Center) (public domain): [http://eros.usgs.gov/#](http://eros.usgs.gov/)

Natural Earth (public domain): <http://www.naturalearthdata.com/>

This is not a copyrighted image. See above.

Reviewers' comments:

Reviewer's Responses to Questions

**Comments to the Author**

1. Is the manuscript technically sound, and do the data support the conclusions?

The manuscript must describe a technically sound piece of scientific research with data that supports the conclusions. Experiments must have been conducted rigorously, with appropriate controls, replication, and sample sizes. The conclusions must be drawn appropriately based on the data presented.

Reviewer #1: Yes

2. Has the statistical analysis been performed appropriately and rigorously?

Reviewer #1: Yes

3. Have the authors made all data underlying the findings in their manuscript fully available?

The [PLOS Data policy](http://www.plosone.org/static/policies.action#sharing) requires authors to make all data underlying the findings described in their manuscript fully available without restriction, with rare exception (please refer to the Data Availability Statement in the manuscript PDF file). The data should be provided as part of the manuscript or its supporting information, or deposited to a public repository. For example, in addition to summary statistics, the data points behind means, medians and variance measures should be available. If there are restrictions on publicly sharing data—e.g. participant privacy or use of data from a third party—those must be specified.

Reviewer #1: Yes

4. Is the manuscript presented in an intelligible fashion and written in standard English?

PLOS ONE does not copyedit accepted manuscripts, so the language in submitted articles must be clear, correct, and unambiguous. Any typographical or grammatical errors should be corrected at revision, so please note any specific errors here.

Reviewer #1: Yes

5. Review Comments to the Author

Please use the space provided to explain your answers to the questions above. You may also include additional comments for the author, including concerns about dual publication, research ethics, or publication ethics. (Please upload your review as an attachment if it exceeds 20,000 characters)

Reviewer #1: This paper provides new data and novel insights into the biology of an invasive ant by providing data on its colony structure in both its native and introduced range. The paper is very well written. The introduction does a very good job putting this work into a general context and the methods are clear allowing the study to be replicated.

Thank you.

All of my suggestions are minor.

Editorial Suggestions:
Line 85-86. Change to “However, few of these species have had their social organization described in both native and introduced populations.”

Done

Line 177. The comma after “Texas” can be deleted.

Done.

Line 121. Change “Not collected” to “missed by this collection method.”?

Done.

Line 208. The first sentence of this section (Aggression scale) is a bit of confusing without more context. “Observations of nest-pairs not included in further analyses were used to adapt standard aggression scales developed to quantify interactions between Argentine ants[34,35] to interactions between N. fulva workers” Can you better explain the context of what you mean / what you are doing here.

I have clarified this section. It now reads, “Standard aggression scales have been developed to quantify intraspecific interactions in Argentine ants (*Linepithema humile*) [34,35]. We conducted preliminary interaction assays to adapt this scale to incorporate the behaviors observed in interactions between *N. fulva* workers. Data from these preliminary assays are not included in the analyses presented herein.”

Line 401. “Stark” can be deleted.

Done.

Line 404-408. This is interesting and worth following up on. The authors could consider adding a graph / table comparing intra- versus inter-specific pattern of aggression to quantify this difference as part of the paper (just a suggestion).

We agree that this is interesting. However, the data for interspecific aggression were collected for a paper on chemical ecology prior to observing intraspecific interactions in the native range and thus prior to the development of this behavioral scale. The qualitative difference is obvious as the interspecific assay was predicated on the ants deploying their chemical defenses rapidly which they did in all interactions (and almost never do in intraspecific interactions). However, these interactions were not scored with the same behavioral scale and thus a direct quantitative comparison is not possible without additional data collection. As this is a related but ancillary issue to the topic of the paper, we are not undertaking this at this time.

Line 424. This paragraph could be better developed in terms of social structure generally in ants. Are there other examples that can be discussed that similar to what is seen here? Are there examples of non-transitive behaviors among colonies. Why do these occur?

 I have added an analysis of why these interactions occur and my suggestions for their significance for studies of ant social structure.

While revising your submission, please upload your figure files to the Preflight Analysis and Conversion Engine (PACE) digital diagnostic tool, <https://pacev2.apexcovantage.com/>. PACE helps ensure that figures meet PLOS requirements. To use PACE, you must first register as a user. Registration is free. Then, login and navigate to the UPLOAD tab, where you will find detailed instructions on how to use the tool. If you encounter any issues or have any questions when using PACE, please email us at [figures@plos.org](mailto:figures@plos.org). Please note that Supporting Information files do not need this step.

I have done so. I saw no way to approve or submit the uploaded figures, so I am assuming that you have access to what I uploaded. I reviewed the figures and they all looked good to me. It was unclear whether you also wanted Tables uploaded in this format, so I went ahead and did so.

I thank you and the Reviewer for your efforts.

Sincerely,

Edward LeBrun
